# Supplementary material for: Deep neurobehavioral phenotyping uncovers neural fingerprints of locomotor deficits in Parkinson’s disease
Source: NPJ Parkinsons Dis. 2026 Feb 7;12:65. doi: 10.1038/s41531-026-01280-4 (PMC12992695; doi:10.1038/s41531-026-01280-4)
Supplement: Supplementary file 1 — Supplementary Information [file 41531_2026_1280_MOESM1_ESM.pdf]

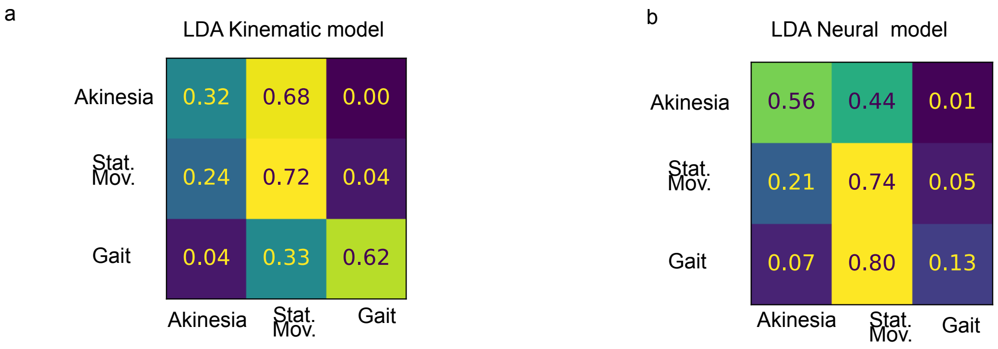

Supplementary Figure 1 Expanded Confusion Matrixes for LDA and CEBRA models a. LDA kinematic model - Confusion Matrix. b. LDA neural model - Confusion Matrix.

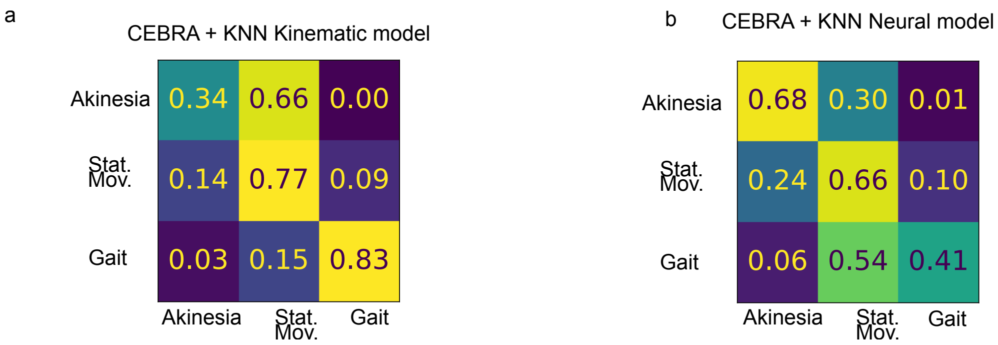

Supplementary Figure 2 Expanded Confusion Matrixes for CEBRA models + KNN a. CEBRA kinematic model - Confusion Matrix. b. CEBRA neural model - Confusion Matrix.

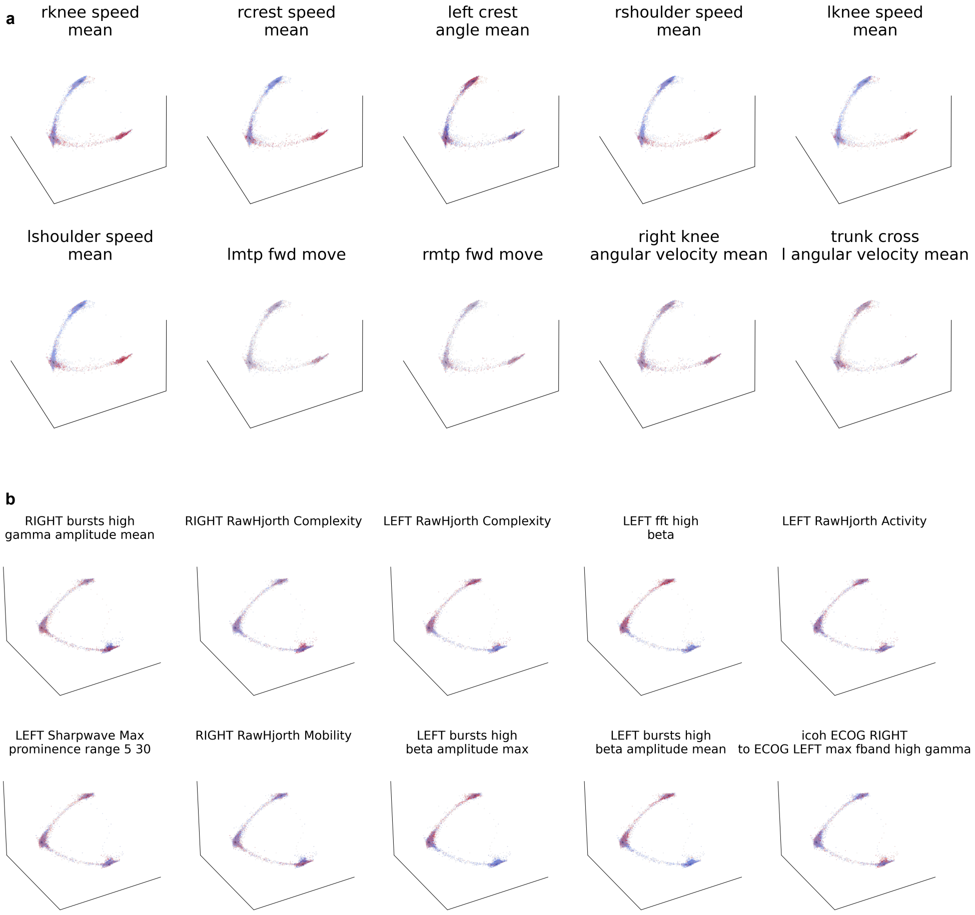

Supplementary Figure 3 Heatmap for top scoring CEBRA features

a. Value distribution of top 10 kinematic features in CEBRA kinematic embedding. b. Value distribution of top 10 neural features in CEBRA neural embedding.

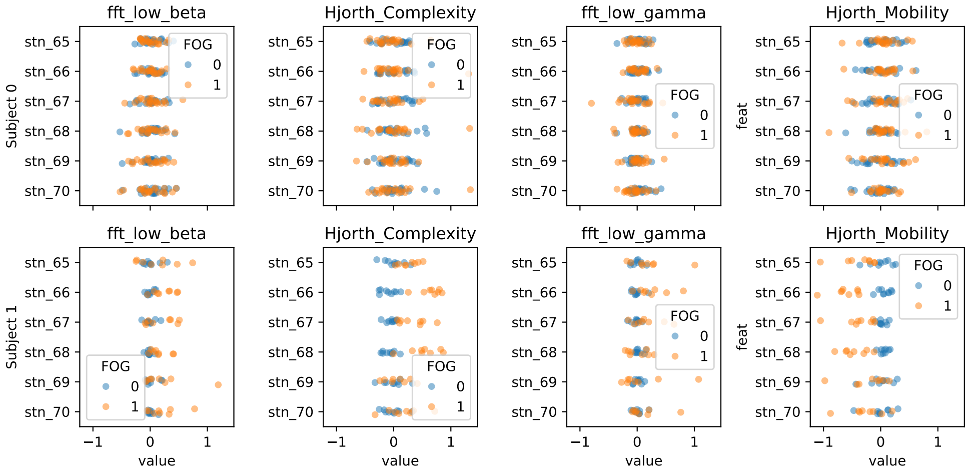

Supplementary Figure 4 Feature distribution in all channels of STN

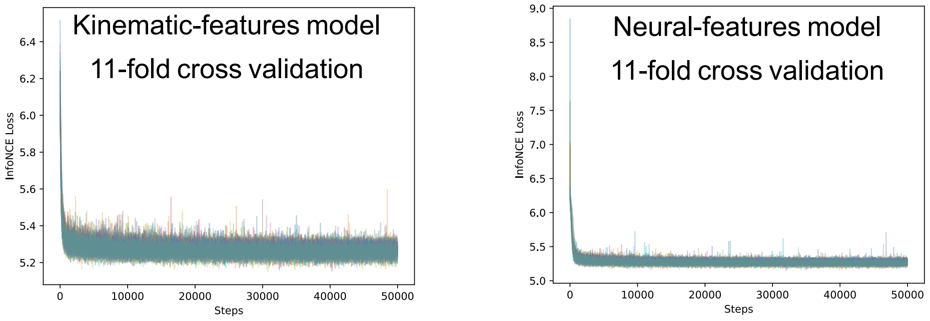

Supplementary Figure 5 Loss function for the kinematic and neural model across 11-fold cross-validation

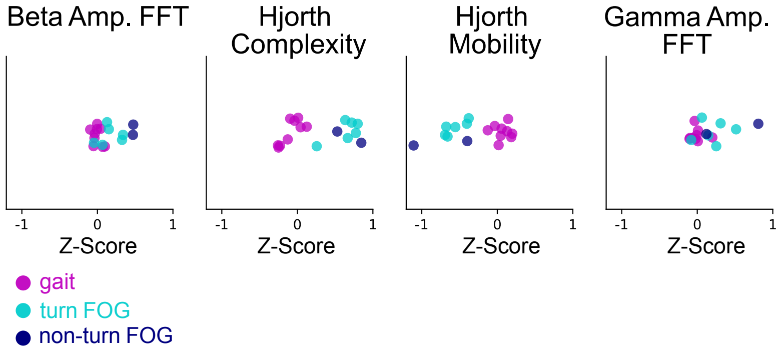

Supplementary Figure 6 **Distribution of feature modulation across type of FOG episodes and gait, for Subject 2**

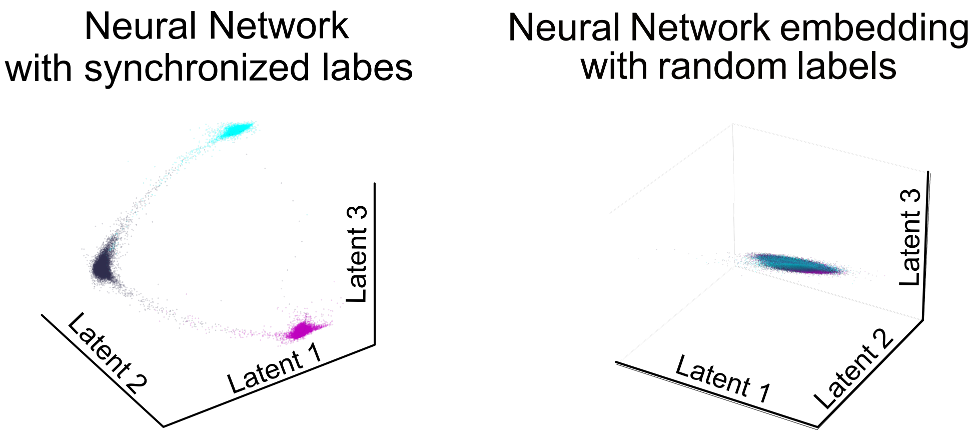

Supplementary Figure 7 **Comparison of embedding conditioned on correct motor states labels vs. collapsed embedding conditioned on shuffled labels**

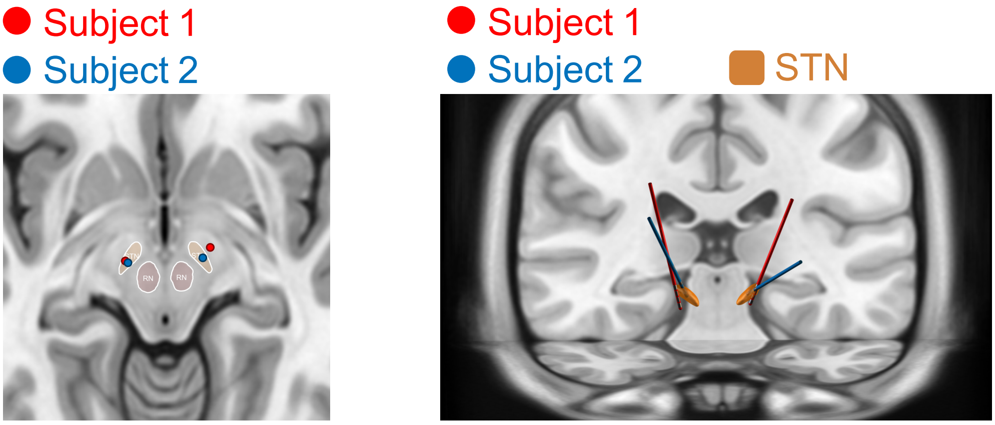

Supplementary Figure 8 **2D slice and 3D reconstruction of electrodes positioning in Subject 1 and 2**

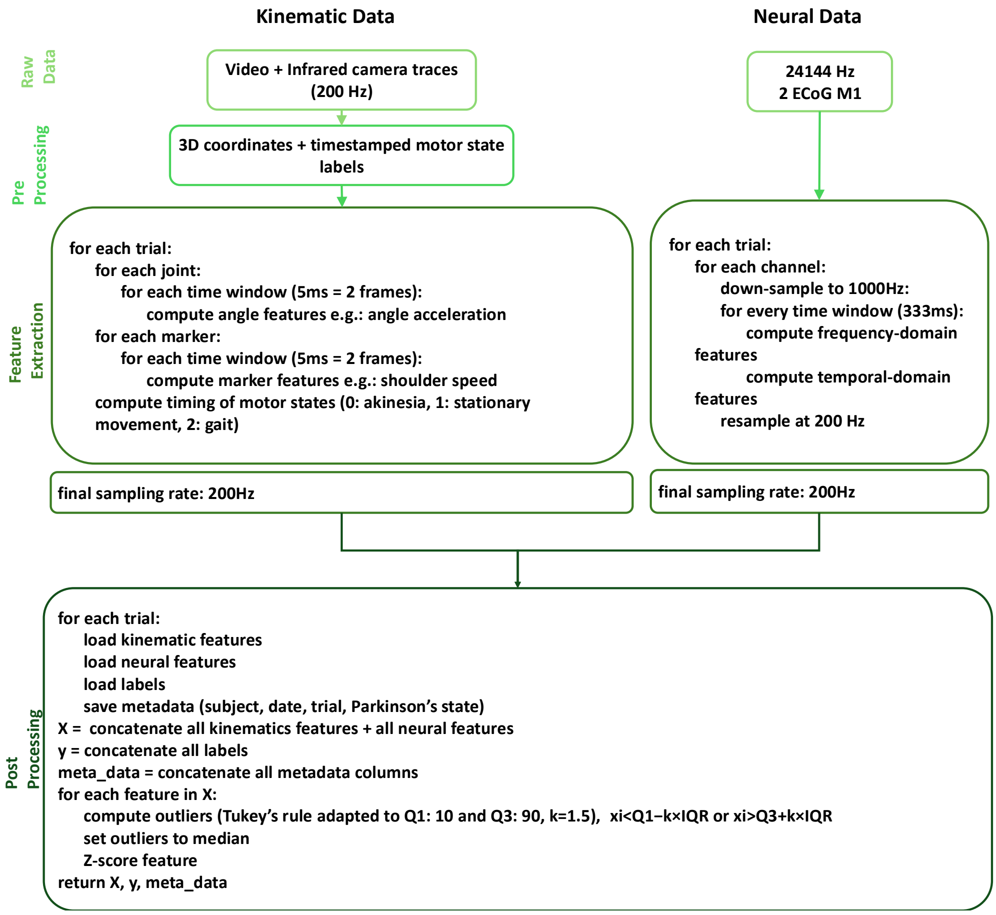

Supplementary Figure 9 Flowchart summarizing the preprocessing steps for the animal data

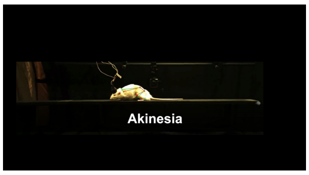

Supplementary Video 1 - Representative labelling of a runway trial of a 6-OHDA lesioned rat, specifically: akinesia, stationary movement and gait.

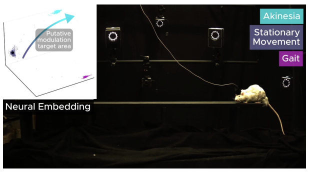

Supplementary Video 2 - Offline neural state tracking during a runway trial of a 6-OHDA lesioned rat. The black moving points tracks the current neural state, moving across the distinct regions.

Feature	Description
left crest angle mean	Mean angle (shoulder-crest-hip) in window period. Left side.
left hip angle mean	Mean angle (crest-hip-knee) in window period. Left side.
left knee angle mean	Mean angle (hip-knee-ankle) in window period. Left side.
left ankle angle mean	Mean angle (knee-ankle-MTP) in window period. Left side.
right crest angle mean	Mean angle (shoulder-crest-hip) in window period. Right side.
right hip angle mean	Mean angle (crest-hip-knee) in window period. Right side.
right knee angle mean	Mean angle (hip-knee-ankle) in window period. Right side.
right ankle angle mean	Mean angle (knee-ankle-MTP) in window period. Right side.
trunk cross l angle mean	Mean angle (left shoulder - right crest - left hip)
trunk cross r angle mean	Mean angle (right shoulder - left crest - right hip)
left crest angular velocity mean	Mean angle velocity (shoulder-crest-hip) in window period. Left side.
left hip angular velocity mean	Mean velocity angle (crest-hip-knee) in window period. Left side.
left knee angular velocity mean	Mean velocity angle (hip-knee-ankle) in window period. Left side.
left ankle angular velocity mean	Mean velocity angle (knee-ankle-MTP) in window period. Left side.
right crest angular velocity mean	Mean angle velocity (shoulder-crest-hip) in window period. Right side.
right hip angular velocity mean	Mean velocity angle (crest-hip-knee) in window period. Right side.
right knee angular velocity mean	Mean velocity angle (hip-knee-ankle) in window period. Right side.
right ankle angular velocity mean	Mean velocity angle (knee-ankle-MTP) in window period. Right side.
trunk cross l angular velocity mean	Mean angle velocity (left shoulder - right crest - left hip)
trunk cross r angular velocity mean	Mean angle velocity (right shoulder - left crest - right hip)
lmtp speed mean	Mean speed of MTP. Left side.
lankle speed mean	Mean speed of ankle. Left side.
lknee speed mean	Mean speed of knee. Left side.
lhip speed mean	Mean speed of hip. Left side.
lcrest speed mean	Mean speed of crest. Left side.
lshoulder speed mean	Mean speed of shoulder. Left side.

Feature	Description
rmtp speed mean	Mean speed of MTP. Right side.
rankle speed mean	Mean speed of ankle. Right side.
rknee speed mean	Mean speed of knee. Right side.
rhip speed mean	Mean speed of hip. Right side.
rcrest speed mean	Mean speed of crest. Right side.
rshoulder speed mean	Mean speed of shoulder. Right side.
lmtp height	Height of MTP. Left side.
lmtp fwd move	Relative forward movement of MTP. Left side.
rmtp height	Height of MTP. Right side.
rmtp fwd move	Relative forward movement of MTP. Right side.
ECOG LEFT RawHjorth Activity	Hjorth Activity. Left side.
ECOG LEFT RawHjorth Mobility	Hjorth Mobility. Left side.
ECOG LEFT RawHjorth Complexity	Hjorth Complexity. Left side.
ECOG RIGHT RawHjorth Activity	Hjorth Activity. Right side.
ECOG RIGHT RawHjorth Mobility	Hjorth Mobility. Right side.
ECOG RIGHT RawHjorth Complexity	Hjorth Complexity. Right side.
ECOG LEFT raw	Raw signal. Left side.
ECOG RIGHT raw	Raw signal. Right side.
ECOG LEFT bandpass activity low beta	Filtered ECoG signal [13, 20] Hz. Left Side.
ECOG LEFT bandpass activity high beta	Filtered ECoG signal [20, 35] Hz. Left Side.
ECOG LEFT bandpass activity low gamma	Filtered ECoG signal [60, 80] Hz. Left Side.
ECOG LEFT bandpass activity high gamma	Filtered ECoG signal [90, 200] Hz. Left Side.
ECOG LEFT bandpass activity HFA	Filtered ECoG signal [200, 400] Hz. Left Side.
ECOG RIGHT bandpass activity low beta	Filtered ECoG signal [13, 20] Hz. Right Side.
ECOG RIGHT bandpass activity high beta	Filtered ECoG signal [20, 35] Hz. Right Side.
ECOG RIGHT bandpass activity low gamma	Filtered ECoG signal [60, 80] Hz. Right Side.
ECOG RIGHT bandpass activity high gamma	Filtered ECoG signal [90, 200] Hz. Right Side.
ECOG RIGHT bandpass activity HFA	Filtered ECoG signal [200, 400] Hz. Right Side.

Feature	Description
ECOG LEFT stft low beta	Short-time Fourier transform [13, 20] Hz. Left Side.
ECOG LEFT stft high beta	Short-time Fourier transform [20, 35] Hz. Left Side.
ECOG LEFT stft low gamma	Short-time Fourier transform [60, 80] Hz. Left Side.
ECOG LEFT stft high gamma	Short-time Fourier transform [90, 200] Hz. Left Side.
ECOG LEFT stft HFA	Short-time Fourier transform [200, 400] Hz. Left Side.
ECOG RIGHT stft low beta	Short-time Fourier transform [13, 20] Hz. Right Side.
ECOG RIGHT stft high beta	Short-time Fourier transform [20, 35] Hz. Right Side.
ECOG RIGHT stft low gamma	Short-time Fourier transform [60, 80] Hz. Right Side.
ECOG RIGHT stft high gamma	Short-time Fourier transform [90, 200] Hz. Right Side.
ECOG RIGHT stft HFA	Short-time Fourier transform [200, 400] Hz. Right Side.
ECOG LEFT fft low beta	Fast Fourier transform [13, 20] Hz. Left Side.
ECOG LEFT fft high beta	Fast Fourier transform [20, 35] Hz. Left Side.
ECOG LEFT fft low gamma	Fast Fourier transform [60, 80] Hz. Left Side.
ECOG LEFT fft high gamma	Fast Fourier transform [90, 200] Hz. Left Side.
ECOG LEFT fft HFA	Fast Fourier transform [200, 400] Hz. Left Side.
ECOG RIGHT fft low beta	Fast Fourier transform [13, 20] Hz. Right Side.
ECOG RIGHT fft high beta	Fast Fourier transform [20, 35] Hz. Right Side.
ECOG RIGHT fft low gamma	Fast Fourier transform [60, 80] Hz. Right Side.
ECOG RIGHT fft high gamma	Fast Fourier transform [90, 200] Hz. Right Side.
ECOG RIGHT fft HFA	Fast Fourier transform [200, 400] Hz. Right Side.
ECOG LEFT Sharpwave Max prominence range 5 80	Maximum prominence of sharpwave in the 5–80 Hz range. Left Side.
ECOG LEFT Sharpwave Mean interval range 5 80	Mean interval of sharpwave in the 5–80 Hz range. Left Side.
ECOG LEFT Sharpwave Max sharpness range 5 80	Maximum sharpness of sharpwave in the 5–80 Hz range. Left Side.
ECOG LEFT Sharpwave Max prominence range 5 30	Maximum prominence of sharpwave in the 5–30 Hz range. Left Side.
ECOG LEFT Sharpwave Mean interval range 5 30	Mean interval of sharpwave in the 5–30 Hz range. Left Side.
ECOG LEFT Sharpwave Max sharpness range 5 30	Maximum sharpness of sharpwave in the 5–30 Hz range. Left Side.
ECOG RIGHT Sharpwave Max prominence range 5 80	Maximum prominence of sharpwave in the 5–80 Hz range. Right Side.
ECOG RIGHT Sharpwave Mean interval range 5 80	Mean interval of sharpwave in the 5–80 Hz range. Right Side.

Feature	Description
ECOG RIGHT Sharpwave Max sharpness range 5 80	Maximum sharpness of sharpwave in the 5–80 Hz range. Right Side.
ECOG RIGHT Sharpwave Max prominence range 5 30	Maximum prominence of sharpwave in the 5–30 Hz range. Right Side.
ECOG RIGHT Sharpwave Mean interval range 5 30	Mean interval of sharpwave in the 5–30 Hz range. Right Side.
ECOG RIGHT Sharpwave Max sharpness range 5 30	Maximum sharpness of sharpwave in the 5–30 Hz range. Right Side.
ECOG LEFT fooof a exp	Exponent of the power spectrum model. Left Side.
ECOG LEFT fooof a offset	Offset of the power spectrum model. Left Side.
ECOG RIGHT fooof a exp	Exponent of the power spectrum model. Right Side.
ECOG RIGHT fooof a offset	Offset of the power spectrum model. Right Side.
ECOG LEFT bursts low beta duration mean	Mean burst duration in the low beta frequency band (13–20 Hz). Left Side.
ECOG LEFT bursts low beta amplitude mean	Mean burst amplitude in the low beta frequency band (13–20 Hz). Left Side.
ECOG LEFT bursts low beta duration max	Maximum burst duration in the low beta frequency band (13–20 Hz). Left Side.
ECOG LEFT bursts low beta amplitude max	Maximum burst amplitude in the low beta frequency band (13–20 Hz). Left Side.
ECOG LEFT bursts low beta burst rate per s	Burst rate per second in the low beta frequency band (13–20 Hz). Left Side.
ECOG LEFT bursts high beta duration mean	Mean burst duration in the high beta frequency band (20–35 Hz). Left Side.
ECOG LEFT bursts high beta amplitude mean	Mean burst amplitude in the high beta frequency band (20–35 Hz). Left Side.
ECOG LEFT bursts high beta duration max	Maximum burst duration in the high beta frequency band (20–35 Hz). Left Side.
ECOG LEFT bursts high beta amplitude max	Maximum burst amplitude in the high beta frequency band (20–35 Hz). Left Side.
ECOG LEFT bursts high beta burst rate per s	Burst rate per second in the high beta frequency band (20–35 Hz). Left Side.
ECOG LEFT bursts low gamma duration mean	Mean burst duration in the low gamma frequency band (60–80 Hz). Left Side.
ECOG LEFT bursts low gamma amplitude mean	Mean burst amplitude in the low gamma frequency band (60–80 Hz). Left Side.
ECOG LEFT bursts low gamma duration max	Maximum burst duration in the low gamma frequency band (60–80 Hz). Left Side.
ECOG LEFT bursts low gamma amplitude max	Maximum burst amplitude in the low gamma frequency band (60–80 Hz). Left Side.
ECOG LEFT bursts low gamma burst rate per s	Burst rate per second in the low gamma frequency band (60–80 Hz). Left Side.
ECOG LEFT bursts high gamma duration mean	Mean burst duration in the high gamma frequency band (90–200 Hz). Left Side.

Feature	Description
ECOG LEFT bursts high gamma amplitude mean	Mean burst amplitude in the high gamma frequency band (90–200 Hz). Left Side.
ECOG LEFT bursts high gamma duration max	Maximum burst duration in the high gamma frequency band (90–200 Hz). Left Side.
ECOG LEFT bursts high gamma amplitude max	Maximum burst amplitude in the high gamma frequency band (90–200 Hz). Left Side.
ECOG LEFT bursts high gamma burst rate per s	Burst rate per second in the high gamma frequency band (90–200 Hz). Left Side.
ECOG RIGHT bursts low beta duration mean	Mean burst duration in the low beta frequency band (13–20 Hz). Right Side.
ECOG RIGHT bursts low beta amplitude mean	Mean burst amplitude in the low beta frequency band (13–20 Hz). Right Side.
ECOG RIGHT bursts low beta duration max	Maximum burst duration in the low beta frequency band (13–20 Hz). Right Side.
ECOG RIGHT bursts low beta amplitude max	Maximum burst amplitude in the low beta frequency band (13–20 Hz). Right Side.
ECOG RIGHT bursts low beta burst rate per s	Burst rate per second in the low beta frequency band (13–20 Hz). Right Side.
ECOG RIGHT bursts high beta duration mean	Mean burst duration in the high beta frequency band (20–35 Hz). Right Side.
ECOG RIGHT bursts high beta amplitude mean	Mean burst amplitude in the high beta frequency band (20–35 Hz). Right Side.
ECOG RIGHT bursts high beta duration max	Maximum burst duration in the high beta frequency band (20–35 Hz). Right Side.
ECOG RIGHT bursts high beta amplitude max	Maximum burst amplitude in the high beta frequency band (20–35 Hz). Right Side.
ECOG RIGHT bursts high beta burst rate per s	Burst rate per second in the high beta frequency band (20–35 Hz). Right Side.
ECOG RIGHT bursts low gamma duration mean	Mean burst duration in the low gamma frequency band (60–80 Hz). Right Side.
ECOG RIGHT bursts low gamma amplitude mean	Mean burst amplitude in the low gamma frequency band (60–80 Hz). Right Side.
ECOG RIGHT bursts low gamma duration max	Maximum burst duration in the low gamma frequency band (60–80 Hz). Right Side.
ECOG RIGHT bursts low gamma amplitude max	Maximum burst amplitude in the low gamma frequency band (60–80 Hz). Right Side.
ECOG RIGHT bursts low gamma burst rate per s	Burst rate per second in the low gamma frequency band (60–80 Hz). Right Side.
ECOG RIGHT bursts high gamma duration mean	Mean burst duration in the high gamma frequency band (90–200 Hz). Right Side.
ECOG RIGHT bursts high gamma amplitude mean	Mean burst amplitude in the high gamma frequency band (90–200 Hz). Right Side.
ECOG RIGHT bursts high gamma duration max	Maximum burst duration in the high gamma frequency band (90–200 Hz). Right Side.

Feature	Description
ECOG RIGHT bursts high gamma amplitude max	Maximum burst amplitude in the high gamma frequency band (90–200 Hz). Right Side.
ECOG RIGHT bursts high gamma burst rate per s	Burst rate per second in the high gamma frequency band (90–200 Hz). Right Side.
coh ECOG RIGHT to ECOG LEFT mean fband low beta	Coherence mean in the low beta frequency band (13–20 Hz) between the right and left sides.
coh ECOG RIGHT to ECOG LEFT max fband low beta	Coherence maximum in the low beta frequency band (13–20 Hz) between the right and left sides.
coh ECOG RIGHT to ECOG LEFT mean fband high beta	Coherence mean in the high beta frequency band (20–35 Hz) between the right and left sides.
coh ECOG RIGHT to ECOG LEFT max fband high beta	Coherence maximum in the high beta frequency band (20–35 Hz) between the right and left sides.
coh ECOG RIGHT to ECOG LEFT mean fband high gamma	Coherence maximum in the high gamma frequency band (90–200 Hz) between the right and left sides.
coh ECOG RIGHT to ECOG LEFT max fband high gamma	Coherence maximum in the high gamma frequency band (90–200 Hz) between the right and left sides.
coh ECOG RIGHT to ECOG LEFT max allfbands high gamma	Coherence maximum across all frequency bands in the high gamma range (90–200 Hz) between the right and left sides.
icoh ECOG RIGHT to ECOG LEFT mean fband low beta	Imaginary coherence mean in the low beta frequency band (13–20 Hz) between the right and left sides.
icoh ECOG RIGHT to ECOG LEFT max fband low beta	Imaginary coherence maximum in the low beta frequency band (13–20 Hz) between the right and left sides.
icoh ECOG RIGHT to ECOG LEFT mean fband high beta	Imaginary coherence mean in the high beta frequency band (20–35 Hz) between the right and left sides.
icoh ECOG RIGHT to ECOG LEFT max fband high beta	Imaginary coherence maximum in the high beta frequency band (20–35 Hz) between the right and left sides.
icoh ECOG RIGHT to ECOG LEFT mean fband high gamma	Imaginary coherence mean in the high gamma frequency band (90–200 Hz) between the right and left sides.
icoh ECOG RIGHT to ECOG LEFT max fband high gamma	Imaginary coherence maximum in the high gamma frequency band (90–200 Hz) between the right and left sides.
icoh ECOG RIGHT to ECOG LEFT max allfbands high gamma	Imaginary coherence maximum across all frequency bands in the high gamma range (90–200 Hz) between the right and left sides.

Supplementary Table 1: **Full list of features used**
